# Antioxidant Profile, Amino Acids Composition, and Physicochemical Characteristics of Cherry Tomatoes Are Associated with Their Color

**DOI:** 10.3390/antiox13070785

**Published:** 2024-06-28

**Authors:** Min Woo Baek, Jong Hwan Lee, Chang Eun Yeo, Su Ho Tae, Se Min Chang, Han Ryul Choi, Do Su Park, Shimeles Tilahun, Cheon Soon Jeong

**Affiliations:** 1 Interdisciplinary Program in Smart Agriculture, Kangwon National University, Chuncheon 24341, Republic of Korea; minwoo100@kangwon.ac.kr (M.W.B.); ljhhh96@kangwon.ac.kr (J.H.L.); xotngh9906@kangwon.ac.kr (S.H.T.);; 2 Department of Horticulture, Kangwon National University, Chuncheon 24341, Republic of Korea; freeum75@gmail.com (C.E.Y.); parkds@kangwon.ac.kr (D.S.P.); 3 Sunmin F&B Co., Ltd., Chuncheon 24341, Republic of Korea; 4 National Institute of Horticultural and Herbal Science, Rural Development Administration, Wanju-gun 55365, Republic of Korea; hanryul192@kangwon.ac.kr; 5 Agriculture and Life Science Research Institute, Kangwon National University, Chuncheon 24341, Republic of Korea; 6 Department of Horticulture and Plant Sciences, Jimma University, Jimma 378, Ethiopia

**Keywords:** anthocyanins, cherry tomatoes, GABA, β-carotene, lycopene, phenolics

## Abstract

This study was conducted to characterize different colored lines of cherry tomatoes and derive information regarding their metabolite accumulation. Different colored cherry tomato cultivars, namely ‘Jocheong’, ‘BN Satnolang’, ‘Gold Chance’, ‘Black Q’, and ‘Snacktom’, were assessed for their firmness, taste characteristics, and nutritional metabolites at the commercial ripening stage. The cultivars demonstrated firmness to withstand impacts during harvesting and postharvest operations. The significant variations in the Brix to acid ratio (BAR) and the contents of phenylalanine, glutamic acid, and aspartic acid highlight the distinct taste characteristics among the cultivars, and the nutritional metabolites are associated with the color of the cultivars. The cultivar choices would be the black-colored ‘Black Q’ for chlorophylls, β-carotene, total flavonoids, and anthocyanins; the red-colored ‘Snacktom’ for lycopene; the orange-colored ‘Gold Chance’ for total phenolics; and the green-colored ‘Jocheong’ for chlorophylls, vitamin C, GABA, glutamic acid, essential amino acids, and total free amino acids. The antioxidant capacity varied among the cultivars, with ‘Gold Chance’ consistently exhibiting the highest activity across the four assays, followed by ‘Snacktom’. This study emphasizes the importance of screening cultivars to support breeding programs for improving the nutritional content and encourages the inclusion of a diverse mix of different colored cherry tomatoes in packaging to obtain the cumulative or synergistic effects of secondary metabolites.

## 1. Introduction

The tomato (*Solanum lycopersicum* L.) originates from South America, where it underwent domestication and improvement primarily in the Andean regions of Ecuador and Peru, which was completed in Mesoamerica [1]. Currently, a diverse range of tomato cultivars exists, with different morphological, physicochemical, and sensory attributes contributing to a wide range of tomato-based foods, either in raw or processed form [2]. In 2021, the global production of tomatoes reached 189.13 million tons, cultivated on 5.17 million ha, with Asia contributing significantly by holding a substantial 63.02% share [3]. Specifically, the Republic of Korea produced 348,983 tons of tomatoes from an area of 5610 ha during the same year [3].

Regular tomato consumption is associated with various health benefits, including anticancer properties, decreased susceptibility to cardiovascular, neurodegenerative, and bowel diseases, and improved immune response, exercise recovery, and skin health [4,5]. Experimental evidence also suggests that tomatoes rich in carotenoids have protective effects against oxidative stress in the retinal pigment epithelium and delay the progression of age-related macular degeneration [6]. These protective effects of tomatoes are primarily linked to their valuable bioactive components, such as β-carotene, lycopene, flavonoids, phenolics, and vitamins C and E, which possess antioxidant properties [5].

The increased amino acid levels in tomatoes play a role in their health-promoting qualities, as amino acids are the building blocks of proteins essential for maintaining cellular structure, facilitating the transport and storage of nutrients, promoting wound healing, and aiding in the repair of damaged tissues [7]. Among the amino acids, γ-aminobutyric acid (GABA) and its precursor, glutamic acid, play signaling roles in a range of tissues [8,9]. GABA-rich tomatoes improve metabolic health as the bioavailability of GABA from pureed tomatoes was found to be similar to that of GABA supplement solution in water [8]. In tomatoes, a high level of GABA was found to accumulate in tomato fruit before the breaker stage and to be catalyzed rapidly thereafter [10]. So, screening cultivars rich in GABA and glutamic acid, which do not show rapid GABA catabolism after reaching the breaker stage, could be advantageous for consumers.

Apart from the above-mentioned antioxidant properties and GABA content, consumer acceptance of tomato fruit relies significantly on the taste and physicochemical attributes like the firmness, color, total soluble solids (TSS), titratable acidity (TA), and the Brix to acid ratio (BAR) [11,12]. The postharvest quality of tomatoes develops during the growth, and the phytochemical composition and antioxidant activity can be affected by agronomic practices, environmental factors such as light intensity, water availability, temperature, and growing media, along with the ripening stages and conditions [4,13,14]. Furthermore, research findings have revealed that the diversity among tomato cultivars affects their physicochemical and antioxidant properties and GABA levels [5,9,15].

This study was conducted in a climate-controlled greenhouse with a hydroponics system under the same agronomic practices based on the hypothesis that the color variation among different cherry tomatoes will reveal significant variations in their metabolite profiles. We used green-colored ‘Jocheong’, yellow-colored ‘BN Satnolang’, orange-colored ‘Gold Chance’, black-colored ‘Black Q’, and red-colored ‘Snacktom’ cherry tomatoes to characterize these different colored lines of cherry tomatoes and derive novel information regarding their metabolite accumulation. Specifically, we expected that a distinct color difference would influence the concentration and composition of metabolites, leading to measurable differences in the nutritional content and antioxidant activity among the cherry tomato cultivars, which could require consuming a mix of different colored cherry tomatoes to benefit from their cumulative effects. In addition, the synergistic effects of all the constituents in tomatoes are likely to surpass the advantages of individual components like lycopene and β-carotene. Thus, this study incorporated both individual parameters and antioxidant activities in four assays to study the combined effect of these individual parameters.

## 2. Materials and Methods

### 2.1. Chemicals

All the chemicals utilized in this study were of analytical reagent grade. HPLC-grade methanol for the mobile phase was purchased from the J.T. Baker chemical company (Center Valley, PA, USA). Potassium dihydrogen phosphate was sourced from Yakuri Pure Chemicals (Kyoto, Japan). Metaphosphoric acid and dimethyl sulfoxide (DMSO) were purchased from Kanto Chemical (Tokyo, Japan). Potassium chloride was procured from Junsei Chemical (Tokyo, Japan). β-carotene, gallic acid, rutin, and ascorbic acid were purchased as standards from Sigma-Aldrich (St. Lous, MO, USA). Additionally, the amino acid standard was purchased from Agilent Technologies (Santa Clara, CA, USA).

The following reagents were also purchased from Sigma-Aldrich (St. Louis, MO, USA): sodium acetate, acetone, ethanol, hexane, Folin–Ciocalteu reagent, sodium carbonate, aluminum nitrate, potassium acetate, DPPH (2,2-diphenyl-1-picrylhydrazyl), ABTS (2,2′-azino-bis(3-ethylbenzothiazoline-6-sulfonic acid)), potassium persulfate, acetate, TPTZ (2,4,6-Tri(2-pyridyl)-s-triazine), hydrochloric acid, ferric chloride, sodium phosphate, potassium ferricyanide, trichloroacetic acid, and iron(III) chloride.

### 2.2. Plant Materials

Five cherry tomato (*Solanum lycopersicum* L.) scion cultivars, namely green-colored ‘Jocheong’ (Taeyang Seed Co., Ltd., Seoul, Republic of Korea), yellow-colored ‘BN Satnolang’, orange-colored ‘Gold Chance’, black-colored ‘Black Q’ (Bunong Seed Co., Ltd., Suwon, Republic of Korea), and red-colored ‘Snacktom’ cherry tomatoes (Pan Pacific Seed Co., Ltd., Yongin, Republic of Korea), were used in this research. Additionally, ‘Bikeio’ rootstock (Bunong Seed Co., Ltd., Suwon, Republic of Korea) was used for this study. The trays for cultivation were arranged within the greenhouse facility of Hoban Agriculture Corporation, situated at coordinates 37°55′29″ N and 127°47′04″ E, at an elevation of 85 m above sea level in Chuncheon, Gangwon Province, Republic of Korea. The seeds were sown on 27 September 2021 on horticultural soil sourced from Pindstrup, Denmark. A 162-hole tray measuring W 280 × L 540 × H 45 mm and a 128-hole tray measuring W 280 × L 540 × H 48 mm, both sourced from Bumnong Co., Ltd. (Jeongup, Republic of Korea), were used for sowing the cherry tomato scion and rootstock seeds, respectively. After sowing, we adequately irrigated the seeded trays through overhead irrigation and then covered them with vermiculite to retain moisture. The germination process took place over 48 h in a germination room under dark conditions, with a controlled temperature ranging between 25 and 28 °C and a relative humidity maintained at 90%. Then, the seedlings were transferred to the greenhouse and grafting was attained within a month of seeding, and the plants had reached a stage of growth suitable for transplantation by 5 December 2021.

The transplanted tomatoes were grown using a hydroponic system within a climate-controlled greenhouse located at coordinates 37°92′ N and 127°75′ E in Gangwon Province, Republic of Korea, during the spring/summer of 2022. The electrical conductivity (EC) of the fertigation solution was regulated within a range of 2.5 to 2.8 deciSiemens per meter (dS m^−1^) while maintaining the pH between 5.5 and 5.8, tailored according to the growth stages. The harvesting commenced on 10 March 2022, with the fruits used for the measurements being harvested on 10 May 2022. Harvesting involved selecting uniform-sized fruits from the third cluster of each plant without physical defects, with the exclusion of fruits located at the tip of the cluster.

After harvesting at the commercial harvesting stage, the fruits were transported to Kangwon National University, where ten representative fruits per cultivar were randomly selected. The physicochemical data were taken the same day and the samples prepared for metabolite analysis were freeze-dried, ground, filtered using a 40 µm filter, and stored in a deep freezer until analysis.

### 2.3. Physicochemical Parameters

Fruits of the five cherry tomato cultivars were measured for the firmness, color values, total soluble solids (TSSs), titratable acidity (TA), and brix to acid ratio (BAR). The fruit firmness at the equator of each fruit was measured following the method described by Baek et al. [16]. This involved applying a maximum force of 10 kg using a rheometer (Sun Scientific Co. Ltd., Tokyo, Japan) fitted with a 3 mm diameter round stainless-steel probe with a flat end at a speed of 1.0 mm/s, and the results were quantified in Newtons (N). The fruit skin color values greenness to redness (a*), blueness to yellowness (b*), and darkness to brightness (L*) were measured according to Baek et al. [16] from ten fruit using a CR-400 Chroma meter (Minolta, Tokyo, Japan). The determination of the TSSs, TA, and BAR values followed the procedure described by Tilahun et al. [13] The TSSs was assessed using an Atago refractometer (Atago Co., Ltd., Tokyo, Japan) at 20 °C, with measurements taken from ten sample fruits. The TA was measured through a Mettler Toledo analyzer (Mettler Toledo Ltd., Zurich, Switzerland). This involved dilution (1 mL juice: 19 mL distilled water) followed by titration until reaching a pH of 8.1, utilizing 0.1 N NaOH. The TA result was quantified as mg 100 g^−1^ citric acid of fresh tomato. The BAR was computed by dividing the TSSs by the TA.

### 2.4. Amino Acids

Three biological replicates of freeze-dried cherry tomato fruit samples (1 g) were extracted using 75% ethanol with ultrasonic extraction for 1 h followed by room temperature extraction for 24 h. After filtering the extract with a 0.2 µm filter, analysis of the amino acid content was conducted using Henderson et al.’s [17] method with a Dionex Ultimate 3000 HPLC. The method utilizes automated online derivatization with o-phthalaldehyde (OPA) for primary amino acids and 9-fluorenylmethyl chloroformate (FMOC) for secondary amino acids, and it involves the separation and detection of amino acids using specific reagents that react with amino groups to form derivatives, which are then quantified by the HPLC system.

### 2.5. Secondary Metabolites

Chlorophylls (Chls) were extracted from the ethanol extract of the cherry tomato cultivars. The Chls extraction process utilized the dimethyl sulfoxide (DMSO) extraction procedure as described by Baek et al. [18]. Absorbance readings were taken at 645 and 663 nm utilizing a microplate reader (SpectraMax ABS Plus, Molecular Devices, Sunnyvale, CA, USA) with a DMSO blank serving as the reference. Then, Chl a, Chl b, and the total Chls were calculated using the equations of Arnon [19], as outlined Tilahun et al. [20].

The total anthocyanin content (expressed as cyanidin-3-glucoside equivalents) was assessed using the pH differential method [21] as described by Tilahun et al. [20]. Freeze-dried radish microgreen samples (0.25 g) were mixed with 5 mL of methanol containing 0.1% HCl. The samples were subjected to ultrasonic treatment three times for 10 min, followed by centrifugation to collect the supernatant. A 50 μL portion of the supernatant was filtered using a 0.45 μm membrane filter (PTFE, 13 mm, Whatman, Maidstone, UK). Subsequently, a mixture of 25 mM potassium chloride buffer (pH 1.0) and 400 mM sodium acetate buffer (pH 4.5) was prepared in 950 μL and developed for 15 min. Readings were taken at 520 nm and 700 nm using a microplate reader (SpectraMax ABS Plus, Molecular Devices, Sunnyvale, CA, USA). Then, the anthocyanin content was calculated using the formula:

Anthocyanin content (mg g^−1^) = (V × A × MW × DF)/(ε × m)

where the variables V, A, MW, DF, ε, and m represent the total volume of the extract (mL), the difference in absorbance values at pH 1.0 and pH 4.5 at 520 nm and 700 nm, the molar mass of cyanidin-3-glucoside (449.2 g mol^−1^), the dilution factor, the molar extinction coefficient in L mol^−1^ cm^−1^ (26,900), and the sample quantity (g), respectively.

The lycopene and β-carotene content of the cherry tomato fruit samples were determined according to the methods described by Tilahun et al. [14]. Freeze-dried tomato samples (1 g) were placed in vials, followed by the addition of 5 mL of ethanol, 5 mL of acetone, and 10.0 mL of hexane to each vial. Subsequently, the vials underwent centrifugation for 15 min at 6000× *g*. Following centrifugation, each vial was supplemented with 3 mL of deionized water, and the samples were subjected to agitation for an additional 5 min. Phase separation was achieved by allowing the vials to stand at room temperature without agitation for 5 min. The hexane layer’s absorbance at 503 and 448 nm, relative to a blank hexane solvent, was measured using a microplate reader (SpectraMax i3, Molecular Devices, Sunnyvale, CA, USA) to determine the lycopene and β-carotene content, respectively. The lycopene content of the samples was subsequently quantified as mg 100 g^−1^ of dry weight following the procedure outlined by Fish et al. [22]

Lycopene content (mg kg^−1^) = (Absorbance at 503 × 0.0312)/kg = (Absorbance at 503 × 31.2)/g

Meanwhile the β-carotene content was determined by comparing the sample readings to the β-carotene standard curve and expressed as mg 100 g^−1^ dry weight of the sample.

The levels of total phenolics and flavonoids in the freeze-dried cherry tomato fruit samples were determined with three replications, following the methodology established in our laboratory, as described by Baek et al. [23]. An ethanolic extract (1 mg mL^−1^) or standard was mixed with 1 mL of 10% Folin–Ciocalteu phenol reagent and 1 mL of 2% sodium carbonate solution for the total phenolics measurement. After 90 min of dark incubation at room temperature, the absorbance was then measured at 750 nm using a microplate reader (Spectramax i3, Molecular Devices, Sunnyvale, CA, USA). The results were expressed as milligrams of gallic acid equivalents per gram of sample (mg GAE g^−1^) after the measurements were compared to the gallic acid calibration curve. For the total flavonoid analysis, 1.5 mL of ethanol, 0.1 mL of 10% aluminum nitrite solution, 0.1 mL of 1 M potassium acetate solution, and 2.8 mL distilled water were combined with an ethanolic extract (1 mg mL^−1^). After stirring, the mixture was left to react for 30 min. The absorbance was then measured at 415 nm using a microplate reader (SpectraMax i3, Molecular Devices, Sunnyvale, CA, USA). The obtained results were compared to a rutin calibration curve and expressed as milligrams of rutin equivalents per gram of sample (mg RE g^−1^).

The vitamin C content was analyzed from the freeze-dried cherry tomato samples in triplicate using reversed phase (RP) liquid chromatography with ultraviolet (UV) detection according to Tilahun et al. [20]. A 1 g sample was combined with 10 mL of 5% metaphosphoric acid (5 g/100 mL) and homogenized for 1 min. Following homogenization, the mixture underwent 10 min centrifugation at 7828× *g*. The liquid layer of the extract was then filtered with 0.22 μm membrane and subjected to analysis using a ZORBAX Eclipse XDB-C18 column (4.6 cm × 250 mm, 5 μm, Agilent Technologies, Santa Clara, CA, USA) coupled with a UV-2075 detector (Jasco, Tokyo, Japan) at 265 nm. The analysis involved a 20 μL injection of 100% MeOH:0.1 M KH_2_PO_4_ (1:9 ratio) at a flow rate of 1 mL min^−1^ as the mobile phase.

### 2.6. Antioxidant Activities

Freeze-dried and ground cherry tomato samples were subjected to extraction following the method previously implemented in our laboratory [23]. The assessment of the Trolox-equivalent antioxidant capacity (ABTS), DPPH radical-scavenging capacity, and ferric-reducing antioxidant power (FRAP) was carried out in triplicate following the procedures described by Baek et al. [23]. Additionally, the reducing power (RP) assay was conducted in triplicate according to the method detailed by Choi et al. [24].

A sample solution (0.2 mL) containing a 10 mg mL^−1^ concentration of the extract was added to a 0.8 mL ethanolic DPPH (0.4 mM) solution. The mixture was allowed to react at room temperature in the dark for 10 min. The absorbance was then measured at 517 nm using a microplate reader (Molecular Devices, Sunnyvale, CA, USA). For the blank, distilled water was used instead of the sample. Then, the calculation for the radical scavenging activity was as follows:

DPPH radical scavenging activity (%) = [1 − (Absorbance of the sample/Absorbance of the blank)] × 100

A stock solution of ABTS was prepared by dissolving it in water to a concentration of 7.4 mM. The cation (ABTS+) was generated by reacting this stock solution with 2.45 mM potassium persulfate and allowing the mixture to stand for 14 h at room temperature in the dark. The ABTS^+^ solution was then diluted with ethanol to achieve an absorbance of 0.70 ± 0.02 at 750 nM. Then, 1.0 mL of diluted ABTS^+^ solution was added to 0.01 mL of sample (10 mg mL^−1^ concentration), and the mixture was left at room temperature for 30 min in the dark. The absorbance was measured at 750 nM using a microplate reader (Molecular Devices, Sunnyvale, CA, USA). For the blank, distilled water was used instead of the sample. Then, the radical scavenging activity was calculated by the following equation:

ABTS radical scavenging activity (%) = [1 − (Absorbance of the sample/Absorbance of the blank)] × 100

The FRAP reagent was prepared fresh daily using 300 mM acetate buffer (pH 3.6), a 10 mM 2, 4, 6-tri (2-pyridyl) -1, 3, 5-triazine (TPTZ) solution in 40 mM HCl, and a 20 mM FeCl_3_·6H_2_O solution in a 10:1:1 (*v*/*v*) ratio. The reagent was warmed to 37 °C in a water bath before use. Then, 0.05 mL of the sample (10 mg mL^−1^ concentration) was mixed with distilled water (0.15 mL) and the FRAP reagent (1.5 mL). The reaction mixture was incubated at 37 °C for 4 min, and the absorbance was measured at 595 nM using a microplate reader (Molecular Devices, Sunnyvale, CA, USA).

For the reducing power (RP) assay, samples (0.1 mL) of 10 mg mL^−1^ concentration were mixed with potassium ferricyanide (1 %, 0.5 mL) and sodium phosphate buffer (0.2 M, 0.5 mL) and incubated at 50 °C for 20 min. Trichloroacetic acid (0.5 mL) was then added to the mixed solution and centrifuged at 1790× *g* for 10 min. In a new test tube, the supernatant (0.5 mL), iron (III) chloride solution (0.1%, 0.1 mL), and distilled water (0.5 mL) were mixed. Then, the absorbance of this solution was measured at 700 nm using a microplate reader (Molecular Devices, Sunnyvale, CA, USA).

### 2.7. Experimental Design and Statistical Analysis

A completely randomized design was used at the greenhouse. To assess the differences between the cherry tomato cultivars, the collected data were subjected to analysis of variance (ANOVA) at *p* < 0.05 using statistical software (SAS/STAT^®^ 9.1; SAS Institute Inc., Cary, NC, USA). Duncan’s multiple range test, heat maps and principal component analysis (PCA) were used to further examine the variations between the cultivars. MetaboAnalyst v6.0 and XLSTAT version 2015.1 (Addinsoft Inc., 244 Fifth Avenue, Suite E100, New York, NY, USA) were used for the heat maps and PCA, respectively.

## 3. Results and Discussion

### 3.1. Physicochemical Parameters

Assessing the firmness of fresh tomatoes is a crucial parameter to evaluate their quality, especially concerning their suitability for the intended culinary uses. Additionally, it serves as a valuable criterion for screening resilient cultivars, ensuring resistance against mechanical injury during harvesting and postharvest operations [16]. In the present study, significant difference in firmness was observed among the five cherry tomato cultivars (Table 1). The highest firmness value was recorded in ‘Black Q’ (17.09 N), followed by ‘Jocheong’ (13.38 N), and the lowest firmness value was recorded in the ‘BN Satnolang’ (10.44 N) cultivar, although the difference between ‘BN Satnolang’, ‘Gold Chance’, and ‘Snacktom’ was not significant. Previous studies have also reported significant differences in firmness among different tomato cultivars [5,13]. The genetic variation in cherry tomato cultivars can influence the cuticle of the tomato fruit, which has been found to have a significant influence on the fruit firmness and ripening physiology, both directly as the cuticle acts as a load-bearing matrix under tension and indirectly by regulating the fruit’s water status [25]. In addition, differences among cultivars may impact the absorption of calcium [26]. An adequate calcium intake is essential for promoting robust growth in tomato plants, reinforcing and stabilizing cell walls, and ultimately, influencing the firmness of the fruit [27].

A significant difference was also observed in the TSSs, TA and BAR of the cherry tomato cultivars (Table 1). The TSSs content of ‘BN Satnolang’ (10.75 °Brix) was the highest and ‘Black Q’ (7.43 °Brix) was the least. The TA values ranged from 0.92 mg 100 g^−1^ in ‘Black Q’ to 1.14 mg 100 g^−1^ in ‘Jocheong’. On the other hand, although the difference between ‘Gold Chance’, ‘Black Q’, and ‘Snacktom’ was not significant, ‘BN Satnolang’ had the highest BAR (11.33), while the lowest BAR (6.84) was observed in ‘Jocheong’ (Table 1), indicating differences in the taste characteristics of the tested cherry tomato cultivars. In agreement with this study, previous studies reported variations in the TSSs, TA, and BAR among cultivars, highlighting that the balance of sugar and acid contents influences the taste characteristics of tomatoes [28,29].

**Figure 1:**
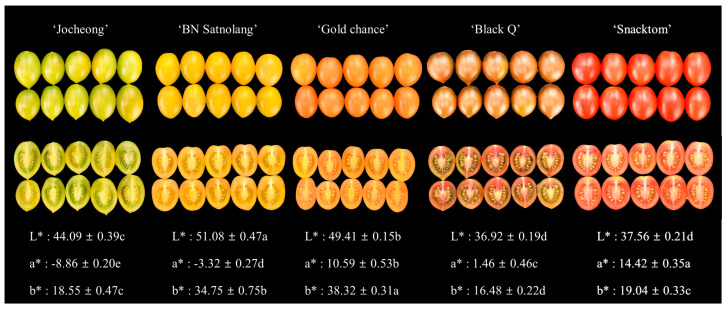
Harvesting stages and external colors of the tested cherry tomato cultivars. The color of the fruit was assessed using the L* (brightness), Hunter a* (redness), and b* (yellowness) values.

The external color of cherry tomatoes is more closely tied to consumer preferences, given its impact on the human eye’s perception of color. Oltman et al. [30] reported color to be the most important external attribute for tomato liking in their survey conducted on consumers’ attitudes and preferences for fresh market tomatoes. Although they reported red-colored tomatoes as attractive, they also stressed that different groups of tomato consumers exist, with specific preferences for health benefits, taste, firmness, and juiciness. In the current study, the color of cherry tomatoes spans from green to red, encompassing a* values that vary from −8.86 in the ‘Jocheong’ to 14.42 in the ‘Snacktom’. The degree of yellowness, represented by the b* values, also ranges from 16.48 in ‘Black Q’ to 38.32 in ‘Gold Chance’, as illustrated in Figure 1.

### 3.2. Amino Acids

In this study, the amino acid content was evaluated among the different colored cherry tomato cultivars tested. A total of 22 free amino acids were identified and the content of amino acids in the cherry tomatoes was significantly dependent on the cultivar (Table 2). The total free amino acid content (TAA) of ‘Jocheong’ was the highest (62.36 g kg^−1^ DW), followed by ‘Black Q’ (52.88 g kg^−1^ DW), ‘Gold Chance’ (35.32 g kg^−1^ DW), ‘Snacktom’ (34.56 g kg^−1^ DW), and ‘BN Satnolang’ (32.05 g kg^−1^ DW), respectively (Table 2). Consistent with the findings of this research, Tilahun et al. [5] tested the ‘TY VIP’, ‘Mamirio’, ‘Tori’, and ‘Arya’ tomato cultivars and also observed variation in the total amino acids, ranging from 38.81 to 57.31 g kg^−1^ DW. The assessment of the protein quality in a food can be performed by examining the levels of nine essential amino acids (EAA), namely methionine, leucine, isoleucine, histidine, tryptophan, valine, phenylalanine, threonine, and lysin [31]. The findings of this study, as indicated in Table 2, revealed the highest essential amino acids content in ‘Jocheong’ (5.96 g kg^−1^ DW), followed by ‘Black Q’ (3.74 g kg^−1^ DW), ‘BN Satnolang’ (2.95 g kg^−1^ DW), ‘Snacktom’ (2.68 g kg^−1^ DW), and ‘Gold Chance’ (2.52 g kg^−1^ DW), respectively. Hence, the contents of EAA in cherry tomatoes is also cultivar-dependent, and having information about the EAA content could assist customers in selecting the cultivar that best suits their preferences.

Among the individual free amino acids, glutamic acid proved to be the most abundant amino acid across all five cultivars. The content varied from 12.05 g kg^−1^ DW (37.62%) for ‘BN Satnolang’ to 27.54 g kg^−1^ DW (52.09%) for ‘Black Q’. Glutamine was the second most abundant free amino acid, except for ‘Black Q’, where the contents of aspartic acid and GABA were higher than glutamine. Generally, GABA, asparagine, aspartic acid, glutamine, and glutamic acid emerged as the predominant amino acids in all five cherry tomato cultivars, constituting a range from 80.78% in ‘Black Q’ to 84.29% in ‘Jocheong’ of the total free amino acids. Consistent with the findings of this study, Tilahun et al. [5] similarly identified these amino acids as the primary constituents, accounting for 80.65 to 89.98% of the total free amino acids in the ‘TY VIP’, ‘Mamirio’, ‘Tori’, and ‘Arya’ tomato cultivars at the pink stage of ripening. While the GABA content in tomatoes reduces significantly during the ripening transition, ‘Kumato’ tomato, which undergoes a color transformation from green to reddish–brown or purple after ripening, has been reported to maintain a high level of GABA [18]. Similarly, in the present study, a high level of GABA (6.22 g kg^−1^ DW) and its precursor, glutamic acid (26.88 g kg^−1^ DW), was recorded in the green-colored ‘Jocheong’, followed by black-colored ‘Black Q’ (4.96 g kg^−1^ GABA and 27.54 g kg^−1^ DW glutamic acid). Hence, when considering the amount of TAA, EAA, and GABA, the preferable cultivars of choice would be the green-colored ‘Jocheong’ and black-colored ‘Black Q’.

Phenolic volatiles, which play a crucial role in shaping our perception of the tomato flavor, encompass a range of compounds derived from the amino acid phenylalanine [11]. Additionally, the umami taste of tomatoes is influenced by the ratio of glutamic acid to aspartic acid, which is essential for the overall taste profile [12]. Notably, the contents of phenylalanine, glutamic acid, and aspartic acid varied significantly among the five cherry tomato cultivars (Table 2), and the ratio of glutamic acid to aspartic acid ranges from 3.54 in ‘BN-Satnolang’ to 5.61 in ‘Snacktom’, underscoring their role in imparting a distinct and unique taste to each cultivar. In the current study, taurine, a naturally occurring sulfur-containing amino acid, was also detected in cherry tomatoes and the amount ranged from 13.66 mg kg^−1^ in ‘BN-Satnolang’ to 31.97 mg kg^−1^ in ‘Black Q’. Taurine, primarily sourced from the seafoods, has attracted significant attention in recent years due to its cardiovascular effects, such as regulating blood pressure, improving cardiac fitness, and enhancing vascular health [32]. Hence, cherry tomato cultivars could also be screened based on their taurine content, and the preferable cultivar based on the amount of taurine would be the black-colored ‘Black Q’.

### 3.3. Secondary Metabolites

Secondary metabolites contribute to the plant’s interaction with its surroundings, enhancing the aroma and pigmentation to attract seed dispersers while serving as a defense mechanism against both biotic and abiotic stresses [31,33]. The positive health impacts of these secondary metabolites obtained from plant foods become apparent through the consumption of various phytochemicals, exhibiting cumulative or synergistic effects [33].

**Figure 2:**
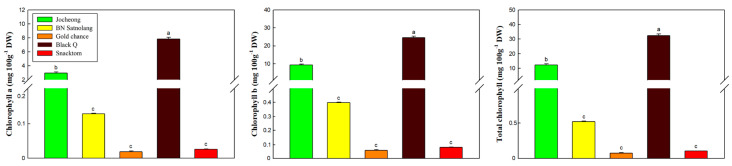
Chlorophyll a, chlorophyll b and total chlorophyll content contents of ‘Jocheong’, ‘BN Satnolang’, ‘Gold chance’, ‘Black Q’, and ‘Snacktom’ cherry tomato cultivars at harvest. Different letters on the bars indicate a significant difference between the mean values of the cultivars (*n* = 3) at *p* < 0.05.

**Figure 3:**
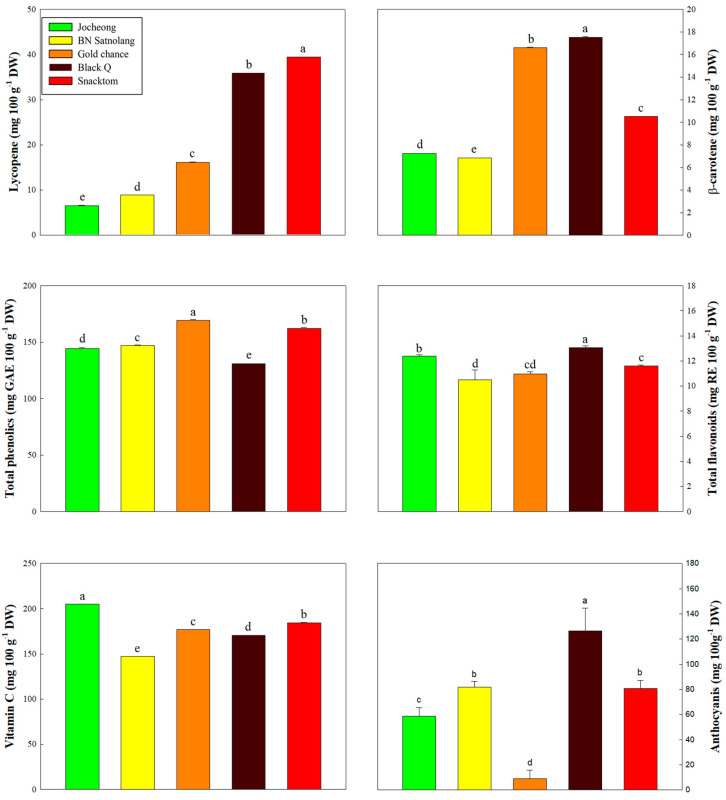
Lycopene, β-carotene, total phenolics, total flavonoids, vitamin C and anthocyanins contents of cherry tomato cultivars at harvest. Different letters on the bars indicate a significant difference between the mean values of the cultivars (*n* = 3) at *p* < 0.05.

The distinct vibrant color of tomato is a result of the transformation of chloroplasts into chromoplasts, a process linked to carotenoid synthesis [14]. As tomatoes ripen, they accumulate lycopene (red linear carotene) and β-carotene (orange cyclization pro-vitamin A product) [34]. This study revealed significant variations among the cherry tomato cultivars in terms of the anthocyanins, chlorophylls, lycopene and β-carotene contents (Figure 2 and Figure 3). In addition to the anthocyanins, the colored tomatoes have the underlining carotenoid pigment, including chlorophylls, lycopene and β-carotene, and these pigments could impact consumers’ preference by indicating maturity, quality and freshness. The green to red (a* values) and blue to yellow (b* values) observed in Figure 1 are directly proportional to the chlorophylls, lycopene and β-carotene contents (Figure 2 and Figure 3). Chlorophylls, lycopene and β-carotene demonstrate significant antioxidant properties, and dietary anthocyanin consumption has been linked to reduced cardiovascular disease risk factors [14,35,36].

In this study, the average total Chls content varied between 0.10 mg g^−1^ DW in ‘Gold Chance’ and 32.40 mg g^−1^ DW in ‘Black Q’ (Figure 2). The concentrations of chlorophyll a, chlorophyll b, and total chlorophylls exhibited similar patterns, with ‘Black Q’ and ‘Jocheong’ showing the highest two scores, respectively. On the other hand, ‘Snacktom’ (39.40 mg 100 g^−1^ DW) and ‘Black Q’ (35.96 mg 100 g^−1^ DW) exhibited the highest lycopene content, followed by ‘Gold Chance’ (16.08 mg 100 g^−1^ DW), ‘BN Satnolang’ (8.87 mg 100 g^−1^ DW), and ‘Jocheong’ (6.54 mg 100 g^−1^ DW) (Figure 3). ‘Black Q’ (17.51 mg 100 g^−1^ DW) had the highest in β-carotene content, followed by ‘Gold Chance’ (16.62 mg 100 g^−1^ DW), while the lowest (7.23 mg 100 g^−1^ DW) was observed in ‘BN Satnolang’ (Figure 3). Similar findings of previous works support the variation in the lycopene and β-carotene contents among cultivars [5,14]. However, ‘BN Satnolang’ and ‘Jocheong’ showed lower levels of lycopene and β-carotene compared to the previously reported ‘TY Megaton’, ‘Yureka’, Tori’, ‘TY VIP’, ‘Mamirio’, and ‘Arya’ tomato cultivars [5,14].

Conversely, ‘Gold Chance’ showed the highest level of total phenolics at 169.36 mg GAE 100 g^−1^ DW, followed by ‘Snacktom’ and ‘BN Satnolang’ with 162.15 and 147.03 mg GAE 100 g^−1^ DW, respectively (Figure 3). The total phenolics content in this study ranged from 144.30 to 169.36 mg GAE 100 g^−1^ DW, slightly lower than the range (168.20 to 290.70 mg GAE 100 g^−1^) reported by Bhandari et al. [37] for Korean commercial tomato cultivars.

The black-colored ‘Black Q’ recorded high levels of total flavonoids (13.05 mg RE 100 g^−1^ DW) and anthocyanins (126.47 mg 100 g^−1^ DW). The green-colored ‘Jocheong’ had a high level of vitamin C (204.92 mg 100 g^−1^ DW), while the red-colored ‘Snacktom’ ranked second in the contents of vitamin C (184.42 mg 100 g^−1^ DW) and anthocyanins (80.60 mg 100 g^−1^ DW) (Figure 3).

Tomatoes, one of the most extensively cultivated vegetables worldwide, are a significant source of bioactive compounds, including carotenoids and polyphenols, such as phenolic acids and flavonoids. Nonetheless, the level of flavonoids in tomatoes is deemed less than optimal, primarily due to the lack of anthocyanins [38]. Therefore, breeding attempts were conducted to develop anthocyanin-enriched tomatoes. Anthocyanin-rich tomatoes developed through breeding programs produce fruits with a dark skin color (purple or black) and high nutraceutical values, combining the health advantages of anthocyanins with those of other tomato phytochemicals, especially carotenoids [39].

Therefore, in the context of secondary metabolites, the preferable cultivar choices would be the black-colored ‘Black Q’ for chlorophylls, β-carotene, total flavonoids and anthocyanins; the red-colored ‘Snacktom’ for lycopene; the orange-colored ‘Gold Chance’ for total phenolics; and the green-colored ‘Jocheong’ for chlorophylls and vitamin C. This suggests the benefit of choosing cultivars tailored to specific target functional compounds and distributing a blend of differently colored cherry tomatoes through packaging for cumulative health benefits.

### 3.4. Antioxidant Activities

Free radicals have been indicated as a natural by-product of aerobic metabolism, where approximately 2–3% of the cell’s oxygen consumption undergoes conversion into these radicals, contributing to the processes of aging and age-related diseases if their amount becomes excessive over an extended period [15,40]. A high intake of fruits and vegetables exhibiting antioxidant activity is receiving increased attention due to their potential in mitigating the detrimental effects of free radicals [15]. The consumption of fresh and processed products made of tomatoes has been reported as a valuable source of antioxidants, contributing significantly to reducing the risk of various cancers [41,42] and plasma lipid peroxidation [15,43]. Therefore, it has become essential to evaluate the nutritional quality of tomatoes by assessing their antioxidant activity. In this study, variations in the antioxidant activity were observed among the five cultivars of cherry tomatoes using four different assays. In all four assays, ‘Gold Chance’ exhibited the highest antioxidant activity, with ‘Snacktom’ ranking second (Table 3). As indicated in Figure 3, ‘Gold Chance’ demonstrated the highest total phenolics, while ‘Snacktom’ showed the highest levels in lycopene and β-carotene. The findings of our study valorize the direct correlations of secondary bioactive metabolites with the antioxidant activity of fresh cherry tomatoes.

### 3.5. Principal Component and Correlation Analysis

**Figure 4:**
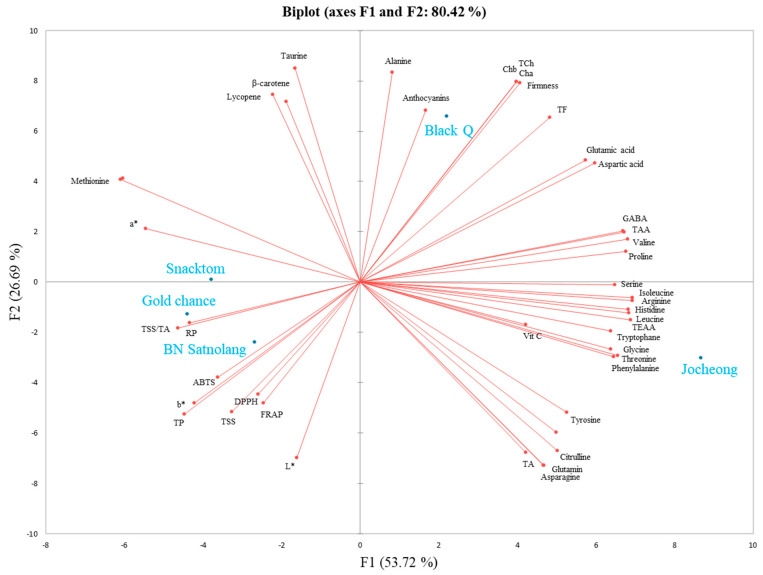
Biplot of nutritional quality parameters in five cherry tomato cultivars at harvest. Data normalization was performed using the median combined with autoscaling, and the analysis was conducted using MetaboAnalyst 6.0 software (https://www.metaboanalyst.ca/ (accessed on 20 November 2023)). The parameters include chlorophyll a (Cha), chlorophyll b (Chb), total chlorophyll (TCh), total soluble solids (TSSs), titratable acid (TA), total amino acids (TAAs), total essential amino acids (TEAs), vitamin C (Vit C), total phenolics (TPs), total flavonoids (TFs), Hunter’s a* (a*), Hunter’s b* (b*), Hunter’s L* (L*), α-diphenyl-β-picrylhydrazyl (DPPH), ferric-reducing antioxidant power (FRAP), 2,2′-azino-bis (3-ethylbenzothiazoline-6-sulfonic acid) (ABTS), and reducing power (RP).

**Figure 5:**
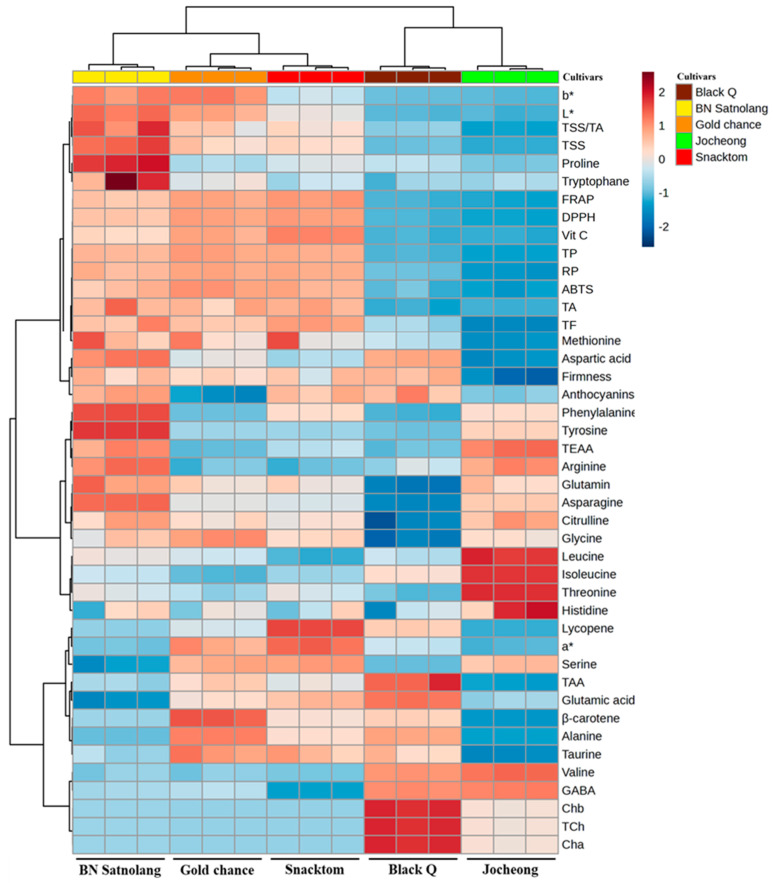
Heat map of the collected parameters for the ‘Jocheong’, ‘BN Satnolang’, ‘Gold chance’, ‘Black Q’, and ‘Snacktom’ cherry tomato cultivars at harvest. Data normalization was performed using the median combined with autoscaling, and the analysis was conducted using MetaboAnalyst 6.0 software (https://www.metaboanalyst.ca/ (accessed on 20 November 2023)). The parameters include chlorophyll a (Cha), chlorophyll b (Chb), total chlorophyll (TCh), total soluble solids (TSSs), titratable acid (TA), total amino acids (TAAs), total essential amino acids (TEAs), vitamin C (Vit C), total phenolics (TPs), total flavonoids (TFs), Hunter’s a* (a*), Hunter’s b* (b*), Hunter’s L* (L*), α-diphenyl-β-picrylhydrazyl (DPPH), ferric-reducing antioxidant power (FRAP), 2,2′-azino-bis (3-ethylbenzothiazoline-6-sulfonic acid) (ABTS), and reducing power (RP).

**Figure 6:**
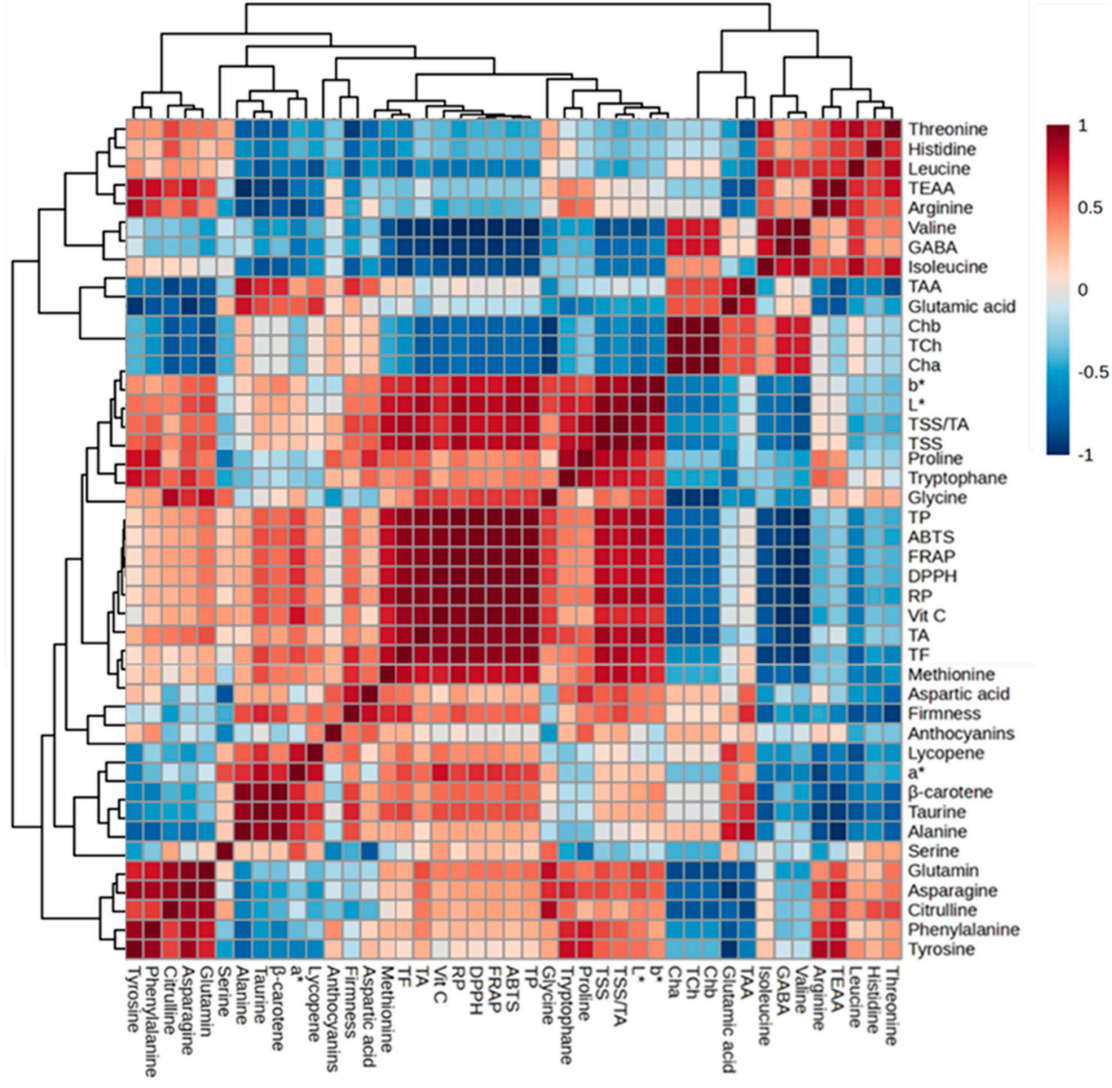
Correlation heat map of nutritional quality parameters in five cherry tomato cultivars at harvest. Data normalization was performed using the median combined with autoscaling, and the analysis was conducted using MetaboAnalyst 6.0 software (https://www.metaboanalyst.ca/ (accessed on 20 November 2023)). The parameters include chlorophyll a (Cha), chlorophyll b (Chb), total chlorophyll (TCh), total soluble solids (TSSs), titratable acid (TA), total amino acids (TAAs), total essential amino acids (TEAs), vitamin C (Vit C), total phenolics (TPs), total flavonoids (TFs), Hunter’s a* (a*), Hunter’s b* (b*), Hunter’s L* (L*), α-diphenyl-β-picrylhydrazyl (DPPH), ferric-reducing antioxidant power (FRAP), 2,2′-azino-bis (3-ethylbenzothiazoline-6-sulfonic acid) (ABTS), and reducing power (RP).

Evaluating the functional properties of tomatoes involves examining their antioxidant activity and determining the key contributors, such as carotenoids, ascorbic acid, phenolics, flavonoids, and anthocyanins. In this study, the different colored cherry tomato cultivars tested were grown under the same agronomic practices based on the hypothesis that the color variation would result in significant differences in their metabolite profiles. In addition, all the parameters were standardized and collected under the same condition for all the cultivars. Consequently, the trends observed in the principal component analysis (PCA) and correlation analysis highlight the parameters that primarily cause variations among the tested cultivars and the relationship between each parameter, respectively. The PCA elucidates the distinctions among treatments and the prominent factors influencing the spatial distribution and correlation of observed parameters. The data collected for cherry tomato cultivars were subjected to the PCA, and the resulting PCA is depicted in Figure 4, illustrating the separation of cultivars based on the observed parameters. The results showed that factor 1 (F1) and factor 2 (F2) together accounted for about 80.42% of the total variance. F1 held the highest variation, elucidating 53.72% of the overall variance, while F2 contributed an additional 26.69% to the total variances. F1 and F2 demonstrated the clear separation of the cherry tomato cultivars, indicating the differences in nutritional quality (metabolites and antioxidant activities) among the five cherry tomato cultivars (Figure 4). Among the five cultivars analyzed, ‘Black Q’ and ‘Snacktom’ showed a high level of lycopene and β-carotene, ‘Gold Chance’ a high level of total phenolics, ‘Black Q’ a high level of total flavonoids and anthocyanins, and ‘Jocheong’ a high level of vitamin C. In addition, the heat map in Figure 5 illustrates the comprehensive distinctions in the physiochemical characteristics, free amino acids, secondary metabolites, and antioxidant activities among the tested cherry tomato cultivars. The correlations among the collected parameters are also shown in Figure 6. Significant positive correlation coefficients were observed between the secondary metabolites (lycopene, β-carotene, vitamin C, total phenolics, flavonoids, and anthocyanins) and the antioxidant activities measured with the four assays (DPPH, FRAP, ABTS, RP) (Figure 6). These results prove the contribution of secondary metabolites to the antioxidant capacity of cherry tomatoes. Moreover, the contribution of the total phenolics to the antioxidant activity was clearly indicated in the PCA for ‘Gold Chance’, ‘Snacktom’, and ‘BN Satnolang’. Our results agree with those of Choi et al. [31], who reported a higher positive correlation of total phenolics with the antioxidant capacity in kiwifruit.

## 4. Conclusions

In this study, five different colored cherry tomato cultivars, namely ‘Jocheong’, ‘BN Satnolang’, ‘Gold Chance’, ‘Black Q’, and ‘Snacktom’, were assessed for their firmness, taste characteristics, and nutritional content. The cultivars exhibited sufficient firmness to withstand impacts during harvesting and postharvest operations, making them well-suited for distribution. The BAR ranged from 11.33 in ‘BN Satnolang’ to 6.84 in ‘Jocheong’, indicating the differences in the taste characteristics of the tested cherry tomato cultivars. A higher amount of a given metabolite means that a smaller quantity of tomatoes is needed to meet the daily requirements, which in turn reduces the production, distribution, and consumption costs. Considering the amount of TAA, EAA, and GABA, the preferred choices were the green-colored ‘Jocheong’ and the black-colored ‘Black Q’. For the secondary metabolites, ‘Black Q’ excelled in β-carotene, total flavonoids, and anthocyanins, while the red-colored ‘Snacktom’ stood out in lycopene. The orange-colored ‘Gold Chance’ led in total phenolics, and the green-colored ‘Jocheong’ topped in vitamin C. The antioxidant activity varied among the cultivars, with ‘Gold Chance’ consistently exhibiting the highest activity across the four assays, followed by ‘Snacktom’. ‘Gold Chance’ also demonstrated the highest total phenolics, while ‘Snacktom’ had the highest levels of lycopene and β-carotene, implying the direct correlation of the secondary bioactive metabolites with the antioxidant activity of fresh cherry tomatoes. In addition, this study recommends selecting cultivars based on the target functional compounds and promoting a diverse mix of colored cherry tomatoes in packaging to align with consumers’ requirements. This approach encourages the consumption of various cultivars, enhancing the cumulative or synergistic effects of secondary metabolites and offering potential health benefits. Future research could explore the findings of sensory acceptance analysis, the impact of various preharvest and postharvest treatments, and the effects of storage on the quality of different colored cherry tomatoes.

## Figures and Tables

**Table 1:** Physicochemical parameters of fresh cherry tomato cultivars at harvest.

Cultivars	Firmness (N)	TSSs (°Brix)	TA (mg 100 g^−1^)	BAR (TSS/TA)
Jocheong	13.38 ± 0.97 b	7.84 ± 0.11 cd	1.14 ± 0.03 a	6.84 ± 0.69 c
BN Satnolang	10.44 ± 0.39 c	10.75 ± 0.20 a	0.96 ± 0.03 b	11.33 ± 1.26 a
Gold chance	10.88 ± 0.52 c	8.66 ± 0.27 b	1.00 ± 0.04 b	8.76 ± 1.34 b
Black Q	17.09 ± 0.60 a	7.43 ± 0.45 d	0.92 ± 0.02 b	8.15 ± 0.64 b
Snacktom	10.82 ± 0.42 c	8.26 ± 0.16 bc	1.01 ± 0.03 b	8.28 ± 0.99 b

TSSs, TA, and BAR stand for total soluble solids, titratable acidity, and brix to acid ratio. The results are shown as the mean ± SD (*n* = 10), with differing letters in the same column indicating significant statistical difference at *p* < 0.05.

**Table 2:** Individual, essential and total amino acid contents in fresh cherry tomato cultivars at harvest.

Amino Acids	Jocheong		BN Satnolang		Gold Chance		Black Q		Snacktom	
	mg kg^−1^	%	mg kg^−1^	%	mg kg^−1^	%	mg kg^−1^	%	mg kg^−1^	%
Aspartic acid	4971.14 b	7.97	3403.79 c	10.62	3254.97 c	9.22	5274.66 a	9.98	3052.74 d	8.83
Glutamic acid	26,883.30 a	43.11	12,054.79 c	37.62	16,446.00 b	46.56	27,544.36 a	52.09	17,118.92 b	49.54
Asparagine	4535.81 a	7.27	2928.94 b	9.14	2108.42 c	5.97	1438.23 e	2.72	2017.01 d	5.84
Serine	1296.15 a	2.08	546.86 d	1.71	729.03 c	2.06	914.87 b	1.73	726.80 c	2.10
Glutamine	9953.29 a	15.96	5992.00 b	18.70	5225.22 c	14.79	3492.97 d	6.61	5069.68 c	14.67
Histidine (EAA)	820.70 a	1.32	347.11 c	1.08	371.14 c	1.05	516.37 b	0.98	364.92 c	1.06
Glycine	108.47 a	0.17	56.25 d	0.18	65.91 b	0.19	68.65 b	0.13	60.04 c	0.17
Threonine (EAA)	1080.68 a	1.73	378.28 cd	1.18	369.27 d	1.05	487.64 b	0.92	401.14 c	1.16
Citrulline	106.72 a	0.17	53.04 b	0.17	50.87 b	0.14	32.31 c	0.06	48.20 b	0.14
Arginine	925.54 a	1.48	483.77 c	1.51	396.16 d	1.12	630.74 b	1.19	385.01 d	1.11
Alanine	607.64 c	0.97	370.36 d	1.16	834.15 b	2.36	1132.56 a	2.14	622.79 c	1.80
Taurine	14.65 c	0.02	13.66 c	0.04	25.90 b	0.07	31.97 a	0.06	23.00 b	0.07
GABA	6220.85 a	9.98	1906.47 d	5.95	2176.56 c	6.16	4960.61 b	9.38	1449.25 e	4.19
Tyrosine	212.93 a	0.34	159.53 b	0.50	77.79 d	0.22	95.15 c	0.18	75.50 d	0.22
Valine (EAA)	342.26 a	0.55	116.12 d	0.36	125.43 c	0.36	263.64 b	0.50	120.33 cd	0.35
Methionine (EAA)	5.74 b	0.01	10.60 a	0.03	10.17 a	0.03	10.29 a	0.02	10.05 a	0.03
Tryptophane (EAA)	366.11 a	0.59	285.02 b	0.89	225.84 c	0.64	282.80 b	0.53	205.82 c	0.60
Phenylalanine (EAA)	1420.66 a	2.28	909.42 b	2.84	621.45 e	1.76	876.84 c	1.66	779.57 d	2.26
Isoleucine (EAA)	677.53 a	1.09	207.36 c	0.65	174.30 d	0.49	384.94 b	0.73	201.38 c	0.58
Leucine (EAA)	640.01 a	1.03	256.19 d	0.80	268.70 c	0.76	385.72 b	0.73	226.90 e	0.66
Lysine (EAA)	609.34 a	0.98	437.21 c	1.36	354.76 d	1.00	532.44 b	1.01	366.47 d	1.06
Proline	558.91 a	0.90	1130.84 b	3.53	1409.95 b	3.99	3519.51 a	6.66	1233.16 b	3.57
Total EAA	5963.04 a	1.29	2947.31 c	2.60	2521.05 d	3.25	3740.67 b	8.11	2676.59 d	2.84
Total	62,358.44 a		32,047.62 d		35,321.97 c		52,877.27 b		34,558.69 c	

EAA stands for essential amino acid. The results are shown as the mean ± SD (*n* = 3), with differing letters in the same raw indicating a significant statistical difference at *p* < 0.05.

**Table 3:** Antioxidant activities of cherry tomato cultivars at harvest.

Cultivars	DPPH (%)	ABTS (%)	FRAP (Absorbance)	RP (Absorbance)
Jocheong	63.69 ± 0.04 c	14.16 ± 0.68 c	0.231 ± 0.002 b	0.222 ± 0.002 c
BN Satnolang	58.55 ± 0.31 d	13.67 ± 0.67 c	0.212 ± 0.003 c	0.221 ± 0.004 c
Gold chance	69.72 ± 0.33 a	16.32 ± 0.09 a	0.250 ± 0.000 a	0.249 ± 0.002 a
Black Q	56.63 ± 0.33 e	13.27 ± 0.71 c	0.202 ± 0.002 d	0.221 ± 0.002 c
Snacktom	69.02 ± 0.31 b	15.22 ± 0.36 b	0.253 ± 0.003 a	0.243 ± 0.001 b

DPPH, FRAP, ABTS, and RP stand for α-diphenyl-β-picrylhydrazyl, ferric-reducing antioxidant power, 2,2′-azino-bis (3-ethylbenzothiazoline-6-sulfonic acid), and reducing power assays, respectively. The results are shown as the mean ± SD (*n* = 3), with differing letters in the same column indicating a significant statistical difference at *p* < 0.05. All the data were detected at 10 mg mL^−1^ methanol extracts of freeze-dried cherry tomatoes.

## Data Availability

The data used to support the findings of this study are included in the article. This study complied with local and national regulations. No collection of seeds or plants is involved in this study.
